# Superior Vena Cava Syndrome in an Infant: A Case of Delayed Diagnosis

**DOI:** 10.7759/cureus.18583

**Published:** 2021-10-07

**Authors:** Stefanie M Miller, Sergio S Cervantes

**Affiliations:** 1 Medicine, University of Central Florida College of Medicine, Orlando, USA; 2 Otolaryngology, Nemours Children's Hospital, Orlando, USA; 3 Otolaryngology, University of Central Florida College of Medicine, Orlando, USA

**Keywords:** pediatric icu procedures, catheter-associated thrombosis, delayed diagnosis, superior vena cava (svc) obstruction, superior vena cava (svc) syndrome

## Abstract

This case presents an infant boy with neonatal respiratory distress and sepsis, who was eventually diagnosed with and treated for superior vena cava (SVC) syndrome after three months of fluctuating head and neck edema. While SVC syndrome is uncommon in pediatrics and is usually caused by malignancy-associated external compression, the growing use of central venous catheters in these patients makes catheter-related thrombosis a potential cause of this serious complication. This case of delayed diagnosis highlights the importance of investigating SVC syndrome as a potential cause of respiratory distress and edema in any patient with a history of central venous catheter placement or similar procedures.

## Introduction

Superior vena cava (SVC) syndrome is a rare occurrence in pediatrics, especially when caused by thrombosis, and must be promptly diagnosed due to the risk of airway obstruction leading to respiratory distress [1,2]. This is a case of a 26-day-old premature male admitted with neonatal respiratory distress and sepsis who developed fluctuating head and neck swelling and continued respiratory difficulty. He was eventually diagnosed with SVC syndrome due to thrombotic occlusion of the vessel, likely as a result of right internal jugular vein central line placement. This case of delayed diagnosis highlights the importance of investigating SVC syndrome in the differential when managing medically complex patients with head and neck edema and respiratory distress, especially with the frequent use of central venous catheters.

## Case presentation

**Figure 1:**
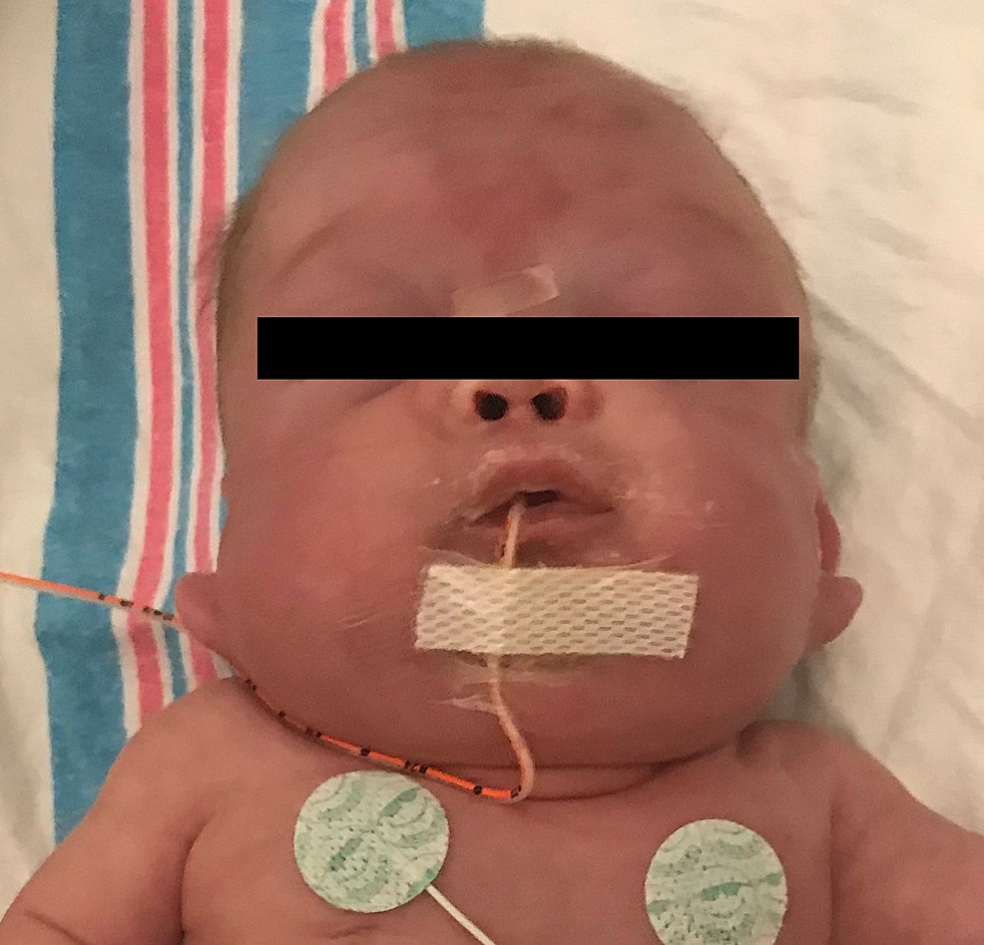
Head and neck edema at three months of age.

A boy born at 24 weeks six days gestation via spontaneous vaginal delivery with a birth weight of 690 grams was admitted to the neonatal intensive care unit (NICU) immediately following delivery due to respiratory distress requiring intubation. NICU stay involved treatment of methicillin-resistant *Staphylococcus aureus* meningitis and sepsis, intraventricular hemorrhage, patent ductus arteriosus, pyogenic arthritis, chronic lung disease of prematurity requiring continuous respiratory support, and retinopathy of prematurity. When the infant was 22 days old, a right internal jugular tunneled central venous catheter was placed via the cutdown technique. Four days later, the patient was noted to have “mild scalp and neck edema” on physical examination. Intermittently over the next few months in the NICU, he was noted to have persistent moderate edema of the face, neck, and scalp (Figure 1). The edema fluctuated in severity but was always present to some degree. He was also noted to have pitting edema of the entire body at times and was managed with chronic diuretics and steroids.

After four months in the NICU, he continued to have difficulty feeding, increased work of breathing, and hypercapnia, which was believed at this point to be due to upper airway obstruction from the edematous airway and possible laryngomalacia, not worsening intrinsic lung disease.

**Figure 2:**
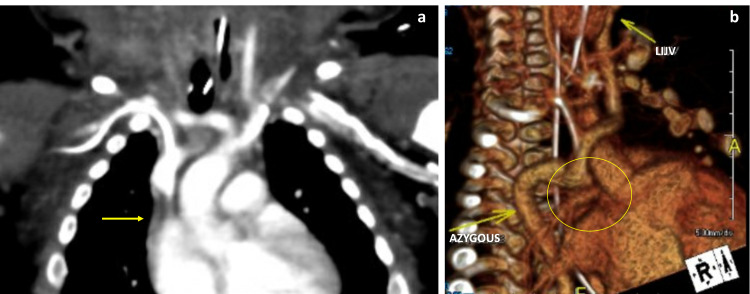
(a) CT angiography showing near-complete occlusion of SVC. (b) CT angiography showing near-complete occlusion of SVC and right internal jugular vein with a large dilated azygous vein and left internal jugular vein (LIJV).

At four months of age, ultrasound of the head and neck soft tissue showed patent bilateral common carotid arteries and jugular veins with diffuse bilateral subcutaneous soft tissue swelling with intervening edema throughout. Due to continued neck swelling and respiratory difficulty, computerized tomography (CT) imaging of the neck soft tissue was performed, which showed complete occlusion of the right internal jugular vein, right subclavian vein, and SVC with marked bilateral neck and face subcutaneous edema. CT angiography of the chest confirmed occlusion of nearly the entire SVC with collateral flow via a large dilated azygous vein (Figure 2).

The patient underwent cardiac catheterization to further evaluate his SVC stenosis and for possible recanalization of the SVC. During attempts to recanalize the SVC, the wire passed into the pericardial space rather than the right atrium. This was recognized prior to the development of a large pericardial effusion. The perforation necessitated the conversion to open surgery via median sternotomy with cardiopulmonary bypass, cardioplegia, and deep hypothermic circulatory arrest. The SVC and right atrium were opened and the area of obstruction was visualized just proximal to the takeoff of the azygous vein. All scar tissue and clot from within the SVC was resected. The perforation was repaired with a pericardium patch placed from the right atrium along the SVC and into the left innominate vein. To reduce intrathoracic pressure until body wall edema was significantly reduced, sternal closure was delayed for five days. He was transported to the ICU post-operatively in stable condition. Post-operative echocardiogram showed flow through the innominate vein and into the SVC with no evidence of obstruction. The patient’s recovery was uneventful apart from sinus node dysfunction, which was managed with pacing as needed to maintain cardiac output and eventually resolved. Head and neck edema gradually resolved afterward, with the improvement of feeding and respiratory difficulties.

## Discussion

SVC syndrome refers to the collection of signs and symptoms caused by occlusion of the SVC, the major vessel for venous drainage from the head, neck, upper extremities, and upper chest [3]. This blockage of blood flow may be partial or complete and results in venous congestion in the upper body, causing swelling of the face, neck, and upper extremity, difficulty breathing, cough, and dilated collateral veins in the chest [1-3]. Swelling of the tissues of the face and neck can be severe enough to cause airway compression and oropharyngeal obstruction [3,4]. SVC obstruction most commonly occurs as a result of compression from a nearby mass, such as mediastinal malignancies, as the vessel is thin-walled and easily flattened [3]. In children, this malignancy is most commonly non-Hodgkin’s lymphoma [1]. An increasing number of cases are being caused by non-malignant, non-compressive etiologies, particularly stenosis or thrombosis from iatrogenic sources, as devices such as pacemaker wires or catheters are being used more frequently [3,4].

In a 2018 review of 142 pediatric cases of SVC syndrome by Nossair et al., 47% were cardiac cases associated with congenital heart disease, 32% were hematologic cases with evidence of SVC thrombus due to risk factors of thrombophilia or central venous catheters, and 31% were oncologic causes associated with leukemia, lymphoma or solid tumors. Across these cases, SVC thrombosis was present in 36% and of these, over 40% had central venous catheters in place [2].

In this patient, the extensive thrombus causing SVC occlusion was likely the result of a right internal jugular central line placed four days prior to the first observation of face and neck edema. However, SVC syndrome was not diagnosed until three months after these first signs of swelling, when the SVC thrombus was noted on neck and chest imaging. Due to the patient’s comorbidities of sepsis and chronic lung disease of prematurity with frequent intubation, the patient’s edema and any worsening of airway status from the SVC syndrome was considered to be a component of his many other serious conditions. As a result, SVC syndrome was not initially investigated, especially as his edema seemed to fluctuate and improve with corticosteroid administration.

SVC syndrome has been noted to cause significant airway obstruction and respiratory distress in pediatric cases [1,2,4]. When occluded, there is a reversal of blood flow in the SVC leading to venous congestion in the smaller tributary veins. Upper airway edema develops due to venous congestion in these vessels, which drain the tongue, posterior oropharynx, and larynx. This leads to engorgement of the mucous membranes and laryngeal edema causes upper airway obstruction [5]. Nossair et al. found that 9% of cases had oropharyngeal obstruction and 10% had acute airway compression, with 67% of cases experiencing dyspnea [2]. Thampi et al. report a case of SVC syndrome in a 40-day-old after bilateral internal jugular vein central line placement that resulted in rapid, severe swelling of the face and neck with cyanosis that required emergent intubation to maintain the airway [4]. Thrombi in the SVC was identified and evacuated surgically.

This patient was successfully treated with surgical recanalization of the SVC with thrombus debridement. Heparin was given in preparation for the procedure. In other cases, anticoagulation, catheterization, or a combination of the two was generally used for treatment. In the review by Nossair et al., 69% of patients with proven SVC thrombosis were treated with anticoagulation, usually unfractionated heparin [2]. For all 142 SVC syndrome cases, 35% underwent catheterization while 27% required open-heart surgery [2].

## Conclusions

Occlusion of the superior vena cava is mostly associated with external compression from malignancy but can result from thrombosis of the vessel, especially in patients with thrombophilia or a history of central venous catheter placement. When the SVC is blocked, the resulting venous congestion in the tributary veins of the oropharynx and larynx can result in significant upper airway edema and obstruction. In a medically complex neonate who has other potential sources of respiratory distress, SVC syndrome may not be considered or easily identified. This case presents an example of delayed diagnosis and highlights the importance of including SVC syndrome in the differential when managing medically complex patients with head and neck edema and respiratory distress, especially with the frequent use of central venous catheters.
